# Risk factor and operation influence of intracranial hemorrhage following ventriculoperitoneal shunt

**DOI:** 10.1038/s41598-025-08799-1

**Published:** 2025-07-15

**Authors:** Wei Wang, Jun Lin, Xiaoming Guo, Shiqi Gao, Junming Zhu, Yuan Hong

**Affiliations:** 1 Department of Neurosurgery, The Second Affiliated Hospital, Zhejiang University School of Medicine, Hangzhou, China; 2 Nursing Department, The Second Affiliated Hospital, Zhejiang University School of Medicine, Hangzhou, China; 3 Clinical Research Center for Neurological Diseases of Zhejiang Province, Hangzhou, China; 4 Key Laboratory of Precise Treatment and Clinical Translational Research of Neurological Diseases, Hangzhou, China

**Keywords:** Intracranial hemorrhage, Ventriculoperitoneal shunt, Complication, Hydrocephalus, Diseases of the nervous system, Medical research, Neurology, Risk factors

## Abstract

Intracranial hemorrhage is a rare but significant complication of ventriculoperitoneal (VP) shunt surgery. This study aimed to identify and assess the risk factors associated with intracranial hemorrhage in patients undergoing VP shunt surgery. This retrospective study included patients who underwent a VP shunt surgery between January 2021 and July 2023. The patients were stratified into two groups based on whether they had a history of prior neurosurgical procedures. Demographic and clinical variables were analyzed for their correlation with postoperative hemorrhage risk. Postoperative CT confirmed hemorrhage. Univariate and logistic regression analyses were performed. Among the 838 patients, 49 (5.85%) developed intracranial hemorrhage. In the overall cohort, preoperative (*P* = 0.037) and postoperative anticoagulant or antiplatelet use (*P* = 0.001), left-sided catheter placement (*P* = 0.026), and pericatheter edema (*P* = 0.052) were associated with hemorrhage. In patients without prior neurosurgery, independent predictors included pericatheter edema (*P* = 0.009), postoperative seizures (*P* < 0.001), shorter catheter depth (*P* = 0.022), and left-sided placement (*P* < 0.001). Postoperative anticoagulant use was significant in patients with prior neurosurgery (*P* = 0.004). Perioperative anticoagulant and antiplatelet use, left-sided catheter placement, and pericatheter edema are critical risk factors for intracranial hemorrhage after VP shunt surgery. Surgical planning should prioritize the vascular anatomy and minimize anticoagulant exposure.

## Introduction

VP shunt surgery is a well-established neurosurgical procedure primarily used to treat hydrocephalus by diverting cerebrospinal fluid (CSF) from the brain ventricles into the peritoneal cavity^1^. Despite its efficacy, it can lead to various complications, including infections, shunt malfunction, and rarely, intracranial hemorrhage^2–5^. Although uncommon, intracranial hemorrhage following VP shunt surgery may result in severe neurological deterioration, seizures, or mortality^6,7^. Identifying the predisposing factors for intracranial hemorrhage is essential for optimizing surgical techniques and postoperative management.

Previous studies have suggested that mechanical factors (e.g., catheter misplacement) and perioperative anticoagulation may contribute to intracranial hemorrhage^8–11^, for instance, history of craniotomy surgery, the useage of anticoagulant drugs and so on. But comprehensive evidence remains limited. This study aimed to systematically evaluate the risk factors of intracranial hemorrhage following VP shunt surgery.

In previous studies, it was found that a history of craniotomy was a risk factor for intracranial hemorrhage after ventriculoperitoneal shunt surgery^8^. Therefore, this study will further distinguish patients based on whether they have a history of craniotomy, in order to investigate the respective risk factors.

This study aimed to investigate the risk factors that may contribute to cerebral hemorrhage following VP shunt surgery. By identifying these factors, we aimed to provide a comprehensive guide for clinicians to reduce the incidence of this complication and enhance the overall safety of VP shunt surgeries.

## Materials and methods

### Patients

This retrospective cohort study included 838 patients who underwent VP shunt surgery at the Second Affiliated Hospital of the Zhejiang University School of Medicine between January 2021 and July 2023. The inclusion criteria were first-time VP shunt placement and postoperative CT imaging within 24 h. All the patients underwent VP shunt surgery through the Kocher’s point approach. The patients were divided into two groups: no prior surgery and prior surgery according to whether the patient had undergone craniotomy before the VP shunt.

**Fig. 1:**
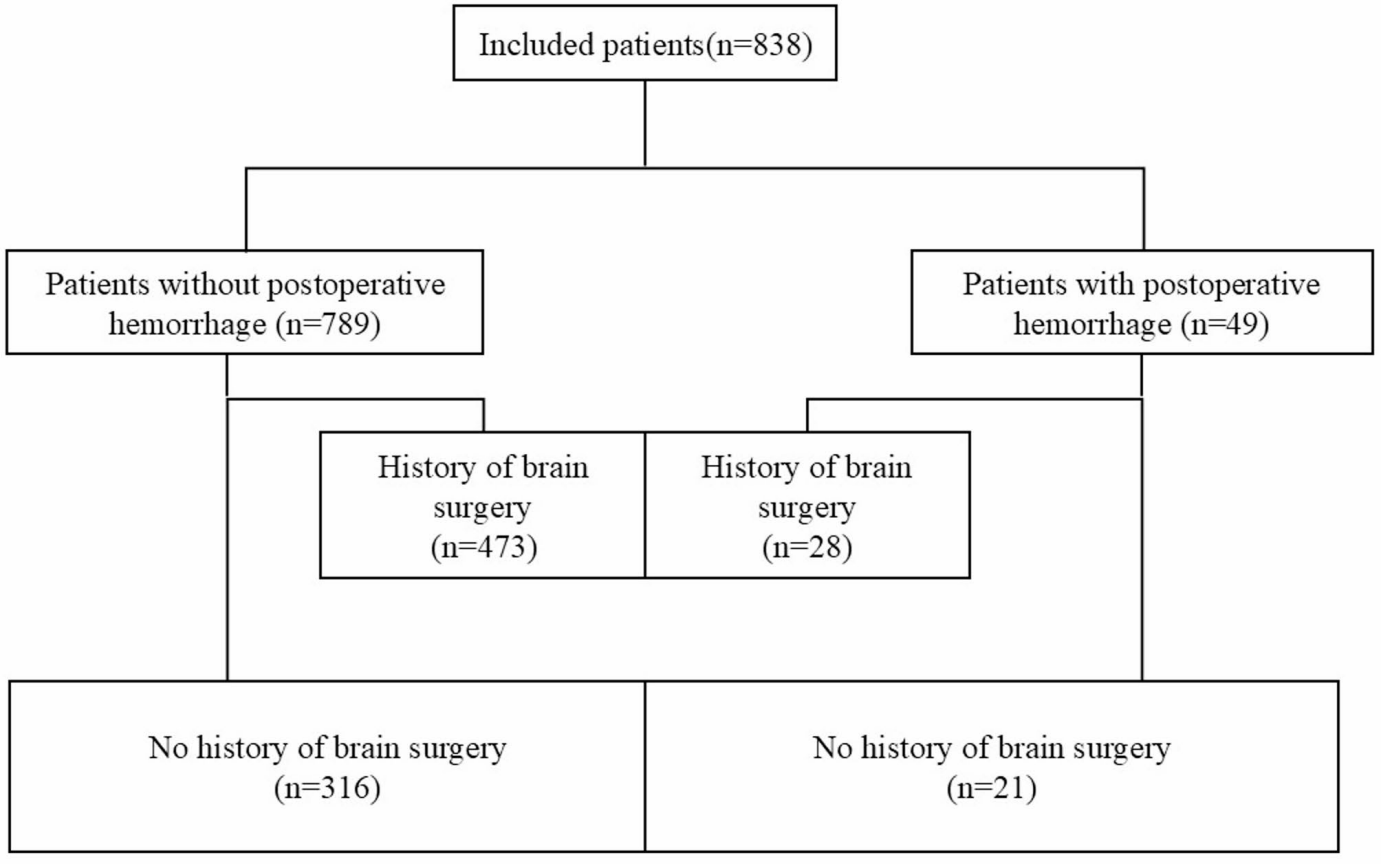
Flow diagram depicting the study.

As Fig. 1 shows, we divided all patients into two groups according to whether they had a history of craniotomy surgery before undergoing VP shunt: no prior surgery and prior surgery. Neurosurgical history was defined as any intracranial procedure before VP shunt placement, including craniotomy, burr hole drainage, decompressive craniectomy, or skull reconstruction. Patients with endovascular interventions (e.g., coiling or embolization) were excluded. No cerebrovascular interventional surgery was performed. This information can be distinguished by medical history or imaging findings prior to surgery. Following surgery, the patients were further divided into two groups based on whether they experienced cerebral hemorrhage, as confirmed by CT imaging: a hemorrhage group and a non-hemorrhage group. Based on the CT images, patients who developed new hematoma near the puncture tract after the surgery, or those with intraventricular hemorrhage, were included in the ICH group. Since it was hard to distinguish whether the intraventricular hemorrhage was primary or the hematoma had entered the ventricle through the puncture tract, both types were included in the same group.

Anticoagulant/antiplatelet agents were discontinued 7 days preoperatively. For patients at high thrombotic risk (e.g., mechanical heart valves), bridging therapy with low molecular weight heparin was administered until 24 h before surgery. The Ethics Committee of the Second Affiliated Hospital of Zhejiang University School of Medicine approved the study protocol, and all patients provided informed consent. All data were collected in accordance with the Declaration of Helsinki.

### Variables and definitions

Several clinical and demographic variables were recorded for each patient, including age, sex, and length of hospital stay. Medical history factors, such as hypertension, diabetes, smoking, alcohol consumption, history of craniotomy, and use of antiplatelet or anticoagulant medications, were also collected.

Preoperative intracranial pressure was assessed using lumbar puncture. Other preoperative data, including preoperative GCS, were also recorded. Postoperative data included the extent of pericatheter edema, seizures, fever, and intracranial infection.

The VP shunt placement was performed via Kocher’s point (located 11 cm posterior to the nasion and 3 cm lateral to the midline) as the standard entry point. Alternative entry points were used in cases of prior craniotomy or anatomical constraints, as documented in surgical records.

**Fig. 2:**
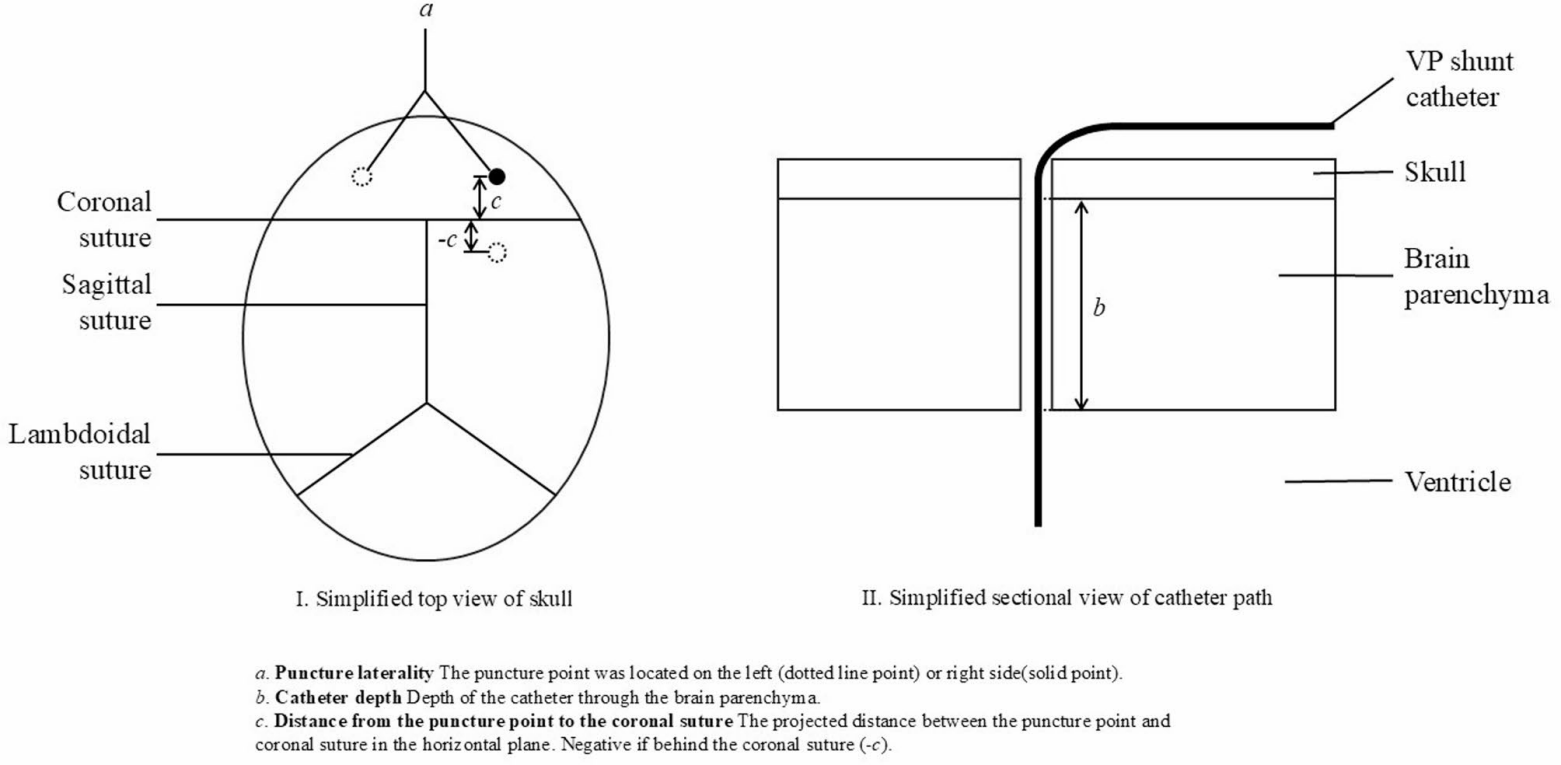
Schematic diagram of operation-related data based on images.

Meanwhile, we collected and measured operation-related data based on postoperative CT images (Fig. 2), as follows:

**Puncture laterality** (left/right): The puncture point of the VP shunt was located on the left or right side.

**Catheter depth**: Depth of the catheter through the brain parenchyma, excluding the part that passes through the skull and into the ventricle.

**Distance from the puncture point to the coronal suture**: The projected distance between the puncture point and coronal suture in the horizontal plane. Positive if the puncture point was in front of the coronal suture and negative behind the coronal suture.

**Pericatheter edema**: Determine the presence of edema around the catheter according to the CT images.

All statistical analyses were performed using SPSS version 22.0 (IBM Corp.). Continuous variables were compared using Student’s t-test or the Mann-Whitney U test, depending on the data distribution. Categorical variables were analyzed using the chi-squared test or Fisher’s exact test. Univariate analysis was conducted, and variables with a P value < 0.1 were included in a binary logistic regression to identify independent risk factors associated with cerebral hemorrhage. Statistical significance was set at *P* < 0.05.

## Results

### Patient and treatment demographics

A total of 838 patients participated in this study from January 2021 to July 2023. The age of the patients was 63 ± 17 years (median ± IQR), with a range of 18 to 87 years. The median length of hospital stay was 12 ± 8 days (range: 3–409 days). Of the 838 samples, 789 patients did not develop cerebral hemorrhage after VP shunt placement and 49 patients developed cerebral or ventricular hemorrhage after surgery. The proportion of patients with postoperative cerebral hemorrhage was 5.85%.

Among those who experienced hemorrhage, 21 had no history of craniotomy surgery. 17 had a history of craniotomy, eight underwent decompressive craniectomy, and three underwent cranioplasty. All patients with a history of surgery were included in the group. In terms of craniotomy history, the rate of cerebral hemorrhage varied: 6.65% in patients with no craniotomy history, 6.94% in those with craniotomy, 5.75% in patients with an open skull, and 3.37% in patients with cranioplasty.

### Univariate and logistic regression analysis

As Table 1 shows, in all samples, factors such as preoperative (*P* = 0.037) and postoperative(*P* = 0.001) use of anticoagulants or antiplatelets, catheter placement location(*P* = 0.026), and the distance between the puncture site and the coronal suture(*P* < 0.001) were significantly associated with cerebral hemorrhage.

**Table 1:** Univariate analysis of variables associated withVP-shunt and ICH.

Variables	Included patients			No prior surgery group			Prior surgery group		
	Non-hemorrhage (*n* = 789)	Hemorrhage (*n* = 49)	P	Non-hemorrhage (*n* = 316)	Hemorrhage (*n* = 21)	P	Non-hemorrhage (*n* = 473)	Hemorrhage (*n* = 28)	P
Demographics									
Sex (Male/Female)	465/324	28/21	0.805	175/141	13/8	0.560	290/15	183/13	0.415
Age, years (Median ± IQR)	63.00 ± 16.00	59.00 ± 17.00	0.153	67.00 ± 16.00	66.00 ± 26.00	0.129	60.00 ± 18.00	55.00 ± 9.00	0.481
Past history									
Hypertension	346	26	0.208	155	10	0.899	191	16	0.080
Diabetes	124	10	0.385	61	7	0.121	63	3	0.692
Smoking history	257	13	0.380	100	6	0.769	157	7	0.369
Drinking history	260	13	0.352	108	5	0.330	152	8	0.694
Preoperative antiplatelet or anticoagulant use	73	9	**0.037**	21	3	0.187	52	6	0.094
Preoperative GCS	11.78 ± 0.14	10.88 ± 0.55	0.124	13.75 ± 0.15	11.90 ± 0.84	**0.042**	10.457 ± 4.164	10.107 ± 3.775	0.665
Image characteristics									
Puncture laterality(Left/Right)	207/582	20/29	**0.026**	22/294	7/14	**< 0.001**	185/288	13/15	0.442
The distance from the puncture point to the coronal suture (mm)	8.306 ± 6.266	12.036 ± 5.927	**< 0.001**	8.15 ± 7.23	11.40 ± 5.72	**0.039**	8.078 ± 6.361	12.445 ± 6.068	< 0.001
Pericatheter edema	293	25	0.052	107	13	**0.009**	186	12	0.710
Catheter depth(mm)	31.32 ± 8.13	29.00 ± 11.12	0.161	30.81 ± 7.70	26.57 ± 11.63	**0.012**	32.001 ± 7.081	31.449 ± 9.396	0.759
Postop. clinical characteristics									
Postoperative GCS	11.39 ± 0.15	10.490 ± 0.57	0.138	13.44 ± 0.16	11.28 ± 0.92	**0.030**	10.011 ± 4.205	9.893 ± 3.804	0.885
Postoperative anticoagulant or anticoagulant use	47	9	**0.001**	15	3	0.060	32	6	**0.004**
Postoperative seizure	42	5	0.150	10	4	**< 0.001**	32	1	0.508
Intracranial infection	76	4	0.734	7	1	0.458	69	3	0.570

After excluding patients with a history of craniotomy surgery, we further analyzed the remaining patients. Pre-(*P* = 0.042) and postoperative(*P* = 0.030) GCS scores, depth of catheter passage through the brain parenchyma(*P* = 0.012), location of the puncture point(*P* < 0.001), distance between the puncture point and coronary suture(*P* = 0.039), pericatheter edema(*P* = 0.009), and postoperative seizure(*P* < 0.001) were correlated with cerebral hemorrhage after VP shunting.

Among patients with a history of craniotomy surgery, the distance from the puncture point to the coronal suture(*P* < 0.001) and postoperative anticoagulant or anticoagulant use(*P* = 0.004) were significantly associated with cerebral hemorrhage.

Logistic regression(Table 2) identified puncture laterality, catheter depth, preoperative and postoperative anticoagulant or antiplatelet use, preoperative and postoperative GCS, pericatheter edema, and postoperative seizures as independent predictors of cerebral hemorrhage in the group with no prior surgery. Notably, left-sided placement of the shunt (*P* = 0.02, OR = 6.302, 95%CI 1.930-20.572), postoperative seizure(*P* = 0.010, OR = 0.128, 95%CI 0.026–0.617) and the short length of the catheter passing through the brain parenchyma(*P* = 0.031, OR = 0.919, 95%CI 0.851–0.992) increased the risk of hemorrhage.

**Table 2:** Logistic regression analysis identifying independent predictive factors of VP shunt and ICH in no prior surgery group.

Variables	No prior surgery group		
	OR	95%CI	*P*
Puncture laterality	6.302	1.930-20.572	**0.002**
Catheter depth	0.919	0.851–0.992	**0.031**
Postoperative seizure	0.128	0.026–0.617	**0.010**
Preoperative antiplatelet or anticoagulant use	0.526	0.084–3.297	0.493
Preoperative GCS	1.010	0.692–1.473	0.960
The distance from the puncture point to the coronal suture	1.024	0.969–1.083	0.394
Pericatheter edema	0.396	0.139–1.127	0.082
Postoperative GCS	0.943	0.655–1.357	0.752
Postoperative anticoagulant or anticoagulant use	3.573	0.056–2.652	0.334

In the group with prior surgery, logistic regression(Table 3) identified pre- and postoperative anticoagulant or antiplatelet use, hypertension, and the distance from the puncture point to the coronal suture. The result comes that the distance from the puncture point to the coronal suture(*P* = 0.043, OR = 1.036, 95%CI 1.001–1.073) and postoperative anticoagulant or anticoagulant use(*P* = 0.050, OR = 0.310, 95%CI 0.096–0.999) are significant.

**Table 3:** Logistic regression analysis identifying independent predictive factors of VP shunt and ICH in prior surgery group.

Variables	Prior surgery group		
	OR	95%CI	*P*
Hypertension	0.504	0.230–1.105	0.087
Preoperative antiplatelet or anticoagulant use	0.756	0.239–2.397	0.635
The distance from the puncture point to the coronal suture	1.036	1.001–1.073	**0.043**
Postoperative anticoagulant or anticoagulant use	0.310	0.096–0.999	**0.050**

In all included patients, lastly, logistic regression(Table 4) identified puncture laterality, pre- and postoperative anticoagulant or antiplatelet use, the distance from the puncture point to the coronal suture and pericatheter edema. Postoperative anticoagulant or anticoagulant use(*P* = 0.028, OR = 0.357, 95%CI 0.142–0.892) is significant.

**Table 4:** Logistic regression analysis identifying independent predictive factors of VP shunt and ICH in all included patients.

Variables	Included patients		
	OR	95%CI	*P*
Puncture laterality	1.805	0.988–3.296	0.055
Preoperative antiplatelet or anticoagulant use	0.664	0.268–1.644	0.375
The distance from the puncture point to the coronal suture	1.023	0.995–1.051	0.103
Pericatheter edema	0.592	0.328–1.068	0.081
Postoperative anticoagulant or anticoagulant use	0.357	0.142–0.892	**0.028**

## Discussion

VP shunt surgery is an essential treatment for hydrocephalus, but it carries the risk of various complications^12^. Infections, shunt obstructions, and subdural hematomas are more commonly reported complications, but cerebral hemorrhage, while rare, poses a serious risk^13,14^. The incidence of cerebral hemorrhage in our study (5.85%) is consistent with previous findings, but slightly higher than earlier reports, such as the 4% rate of delayed hemorrhage reported by Savitz and Bobroff^15^. The differences in incidence rates may be due to differences in surgical techniques, patient selection, or postoperative monitoring protocols.

The mechanism of cerebral hemorrhage after VP shunting has not yet been fully elucidated. Khandelwal et al. suggested that diffuse endovascular coagulation may be the cause of cerebral hemorrhage after VP shunt^16^. Okazaki et al. suggested that congenital CNS abnormalities and respiratory problems cause cerebral hemorrhage after a VP shunt^17^.

However, our findings revealed that the choice of puncture site significantly affected postoperative cerebral hemorrhage. First, we confirmed that performing VP shunting (VPS) on the left side was a risk factor for delayed postoperative hemorrhage. This may be because performing VPS on the patient’s dominant hemisphere increases the risk of cerebral hemorrhage^18^. Second, the distance between the puncture site and coronal suture is also an important factor influencing the risk of postoperative cerebral hemorrhage. Specifically, in reference to the coronal suture, the probability of hemorrhage was lower for puncture sites located farther from the suture, suggesting that we need to improve the accuracy of catheter puncture to avoid regions with a high density of blood vessels. Selecting a more anterior puncture site may increase the distance to the ventricle, which could explain why the risk of hemorrhage is lower in patients without surgical history when the puncture path through the brain parenchyma is longer.

Our findings corroborate previous reports indicating that left-sided shunt placement significantly increases the risk of hemorrhage. Cui Y et al. consider that there is a correlation between lateralization of cerebral basal ganglia hemorrhage and handedness. This means that right-handers are more likely to have a hemorrhage on the left side of the brain and vice versa^18^. They also found that the mean blood flow velocity in the right middle cerebral arteries of most left-handed individuals was relatively higher, and the mean blood flow velocity in the left middle cerebral arteries of most right-handed individuals was relatively higher. This is consistent with our findings. We believe that the difference in the bilateral bleeding risk during VP shunt surgery is due to the same reason. Left-sided catheter trajectories may traverse regions with higher vascular density (e.g., perisylvian arteries) in the dominant hemisphere, thereby increasing the risk of inadvertent vessel injury. On the other hand, the influence of the surgeon on the surgery cannot be ignored either. In most cases, the surgeons are right-handed, which leads to more errors when performing VPS on the left side, such as failed puncture or repeated punctures. The surgeons involved in this study were all right-handed. Performing procedures on the left side of the head can be technically more challenging and less ergonomic. This also reminds us that when performing the surgery on the left side, we should be even more cautious to prevent the occurrence of hemorrhage incidents. In conventional surgery, the right side is always selected as the surgical approach for VP shunting. However, in some cases, a left VP shunt is chosen because it has left non-communicating hydrocephalus or lesions on the right side that are deemed unsuitable for surgery. Surgeons must carefully weigh the risk of bleeding.

Additionally, the proximity of the puncture site to the coronal suture also appears to influence the hemorrhage risk, with more anterior placements associated with higher rates of ICH. Yamada et al. recommended using the right parieto-occipital approach for VP shunt placement to reduce the risk of hemorrhage^19^. Baregzai Y et al. found that the puncture point of Keen’s is more likely to result in a lower revision rate compared to Kocher’s puncture point^20^. Essentially, more posterior puncture point is a better choice. The parieto-occipital approach may also reduce the risk of hemorrhage after the VP shunt because it will reduce head rotation. Therefore, we suggest that, when selecting the puncture point during surgery, the catheter should not be too close to the frontal lobe. Coronal suture is an important sign of the skull, but it cannot be located by the naked eye before surgery because of scalp obstruction. Therefore, it is recommended that the surgeon first locate the location using imaging, such as CT, before the operation.

Pericatheter edema, another independent risk factor, may be related to increased intracranial venous pressure or direct damage to the small vessels during catheter placement. Edema around the catheter may exacerbate the risk of ICH, particularly in patients with fragile vascular structures. Chen JC et al. have proposed that catheter insertion may lead to a disturbance in venous return or hemostasis of a cortical vein and then contribute to subcortical hemorrhage^21^. Brain edema around the catheter is regarded as a radiographic sign of vascular erosion and can be used to predict ICH. Our results are consistent with those of previous studies. We also considered that as the water around the catheter recedes, bleeding occurs in some of the damaged vessels that were originally compressed.

However, studies have shown that postoperative intracranial hemorrhage correlates with the distance of the catheter through the brain parenchyma. Catheters in the ICH group had a shorter distance through the brain parenchyma than those without ICH. We consider this to be due to different degrees of hydrocephalus pressing on the brain parenchyma. As with ICH due to pericatheter edema, highly compressed brain parenchyma is released after surgery due to pressure, increasing the risk of bleeding. The greater the degree of compression of the brain parenchyma, the shorter the distance of the catheter through the brain parenchyma. Surgeons need to carefully assess the degree of compression of the brain parenchyma before surgery to avoid ICH.

One of the most significant findings of our study was the strong association between perioperative anticoagulant or antiplatelet use and postoperative hemorrhage. While anticoagulant therapy is necessary for patients with comorbid conditions, such as atrial fibrillation or a history of thromboembolism, our results suggest that the use of these medications increases the likelihood of hemorrhage. Kandula V et al. study found no significant association between low molecular weight heparin use and deep vein thrombosis in patients after surgery^22^. However, our study found an association between anticoagulant use after surgery and ICH after VP shunt placement. Previous studies have shown that the use of heparin can prevent postoperative patients from developing deep vein thrombosis. However, some other studies have found that this treatment may be ineffective^23^. Therefore, more attention should be paid to the risk of bleeding caused by anticoagulants after surgery. Careful perioperative management, including timely discontinuation of anticoagulants and close monitoring of coagulation status, is essential to mitigate this risk.

Postoperative seizures were also strongly correlated with hemorrhage, suggesting that seizures may be both a consequence and a cause of hemorrhage. Seizures can lead to sudden increases in intracranial pressure, causing vascular rupture and hemorrhage, particularly in patients with preexisting vascular fragility.

The results of this study provide valuable insights into the management of patients who undergo VP shunt surgery. Surgeons should carefully evaluate the risk of hemorrhage, particularly in patients with a history of anticoagulant or antiplatelet use. In addition, selecting the puncture site based on anatomical considerations, such as avoiding the dominant hemisphere and minimizing the distance from the coronal suture, may reduce the risk of postoperative hemorrhage. Postoperative monitoring should be particularly vigilant in patients who experience seizures or present with pericatheter edema.

The present study has several limitations. First, this was a retrospective study that used multivariate analysis to minimize patient selection bias. Second, the inclusion criteria of the samples in this study were relatively lenient, and patients with different surgical histories were not analyzed in more detail. Third, the prognosis and long-term follow-up of patients should be studied.

## Conclusion

In conclusion, perioperative anticoagulant use, left-sided catheter placement, and pericatheter edema are key modifiable risk factors for intracranial hemorrhage following VP shunt surgery. Surgeons should prioritize vascular anatomy during trajectory planning and adopt individualized anticoagulation protocols. Future studies should integrate advanced imaging for real-time vascular mapping to optimize surgical safety.

## Data Availability

The raw data supporting the conclusions of this article will be made available by the authors without undue reservation. One can contact J.L. to request the data from this study.
